# Comprehensive analyses of how tubule occlusion and advanced glycation end-products diminish strength of aged dentin

**DOI:** 10.1038/srep19849

**Published:** 2016-01-22

**Authors:** Yuko Shinno, Takuya Ishimoto, Mitsuru Saito, Reo Uemura, Masumi Arino, Keishi Marumo, Takayoshi Nakano, Mikako Hayashi

**Affiliations:** 1 Department of Restorative Dentistry and Endodontology, Osaka University Graduate School of Dentistry, 1-8 Yamadaoka, Suita 565-0871, Japan; 2 Division of Materials and Manufacturing Science, Osaka University Graduate School of Engineering, 1-2 Yamadaoka, Suita 565-0871, Japan; 3 Department of Orthopaedic Surgery, The Jikei University School of Medicine, 3-25-8 Nishi-shinbashi, Minatoku, Tokyo 105-0003, Japan

## Abstract

In clinical dentistry, since fracture is a major cause of tooth loss, better understanding of mechanical properties of teeth structures is important. Dentin, the major hard tissue of teeth, has similar composition to bone. In this study, we investigated the mechanical properties of human dentin not only in terms of mineral density but also using structural and quality parameters as recently accepted in evaluating bone strength. Aged crown and root dentin (age ≥ 40) exhibited significantly lower flexural strength and toughness than young dentin (age < 40). Aged dentin, in which the dentinal tubules were occluded with calcified material, recorded the highest mineral density; but showed significantly lower flexural strength than young dentin. Dentin with strong alignment of the *c*-axis in hydroxyapatite exhibited high fracture strength, possibly because the aligned apatite along the collagen fibrils may reinforce the intertubular dentin. Aged dentin, showing a high advanced glycation end-products (AGEs) level in its collagen, recorded low flexural strength. We first comprehensively identified significant factors, which affected the inferior mechanical properties of aged dentin. The low mechanical strength of aged dentin is caused by the high mineral density resulting from occlusion of dentinal tubules and accumulation of AGEs in dentin collagen.

In clinical dentistry, since fracture is a major cause of tooth loss, better understanding of mechanical properties of dentin, the main constituent of human teeth, is important. By weight, dentin comprises approximately 70% inorganic material, 20% organic material, and 10% water, and mostly consists of hydroxyapatite and type 1 collagen^1^,^2^. Microstructural features of dentin are dentinal tubules, peritubular dentin and intertubular dentin. Dentinal tubules with an internal diameter of approximately 1 ~ 5 μm are oriented radially from the pulp toward the dentin-enamel junction. Each tubule is surrounded by a highly mineralized cuff of peritubular dentin^1^,^3^. Intertubular dentin occupies the internal space between the peritubular cuffs and consists of mineralized collagen fibrils arranged in a felt-like structure oriented perpendicular to the tubules^4^.

The mechanical strength of human dentin has been investigated by various structural aspects. The mechanical strength of aged dentin is significantly lower than that of young dentin^5^,^6^,^7^,^8^. Dentin is known to change form to transparent (or sclerotic) dentin with aging because the tubule lumens gradually were filled with minerals^1^,^9^,^10^,^11^. This alteration usually begins at the apical end of the root and often extends into the coronal dentin^1^,^12^. Webber *et al*.^13^ claimed that up to 50% of the dentinal tubules can become completely occluded with age under natural physiological conditions. Accordingly, the mineral density of transparent root dentin was significantly higher than normal dentin and its fracture toughness was 20% lower^6^. However, it is not clear whether occluded tubule lumens alone or any other alterations in intertubular dentin are responsible for the low fracture strength of aged dentin^14^,^15^,^16^,^17^.

Tubule orientation has also been recognized as an important factor influencing the mechanical properties of dentin^18^,^19^,^20^,^21^,^22^,^23^,^24^. The flexural and tensile strength of dentin shows anisotropy in terms of dentinal tubule orientation^18^,^19^,^20^,^21^,^22^. Dentin with tubules orientated perpendicular to the long axis and short axis of beam-shaped specimens had significantly higher flexural and tensile strength than dentin with tubules orientated parallel to their long axis. Further investigations into the mechanical properties of dentin showed that dentinal tubule density on the enamel side (11,000–30,000 tubules/mm^2^) was significantly lower than on the pulp side (50,000–70,000 tubules/mm^2^), and that the flexural and tensile strength of dentin on the enamel side was significantly higher than on the pulp side^18^,^19^.

Regarding organic components, Miguez *et al*.^25^ demonstrated the significant contribution of collagen to the mechanical properties of dentin. Levels of two major enzymatic cross-links (dihydroxylysinonorleucine and pyridinoline) in root dentin were significantly higher than those in crown dentin. The authors explained that this contributed to the higher tensile strength of root dentin compared with that of crown dentin. Açil *et al*.^26^ examined the contents of deoxypyridinoline and pyridinoline in dentin in relation to aging and showed that neither cross-links increased with aging. Non-enzymatic cross-links, known as advanced glycation end-products (AGEs) have also been investigated. Miura *et al*.^27^ confirmed by immunohistochemical analysis that one AGE, *N*-carboxymethyllysine (CML), accumulated in dentin collagen with aging.

However, most previous studies investigated the mechanical properties of dentin in relation to a single factor or a limited number of factors. Few studies have comprehensively analysed the mechanical properties of dentin in relation to multiple factors^5^,^6^. Therefore, the dominant elements affecting the mechanical properties of dentin are not fully understood. We speculated that dynamic analysis of bone could offer lessons for dentin.

Bone is hard tissue and has a similar composition to dentin^1^,^2^. Bone mineral density (BMD) is correlated with bone strength^28^. However, the National Institutes of Health’s consensus statement about osteoporosis states that BMD is not an absolute parameter accounting for all of bone’s mechanical properties. Instead, bone strength should be determined by two features: BMD and bone quality^29^. BMD is expressed as grams of mineral per area or volume, while bone quality refers to the architecture, turnover, and damage accumulation such as microfractures and mineralization. To understand bone mechanical function properly, bone strength has been evaluated in terms of both structural and quality parameters^30^. Structural parameters include microstructures such as cancellous bone trabeculae and the porosity of cortical bone, while quality parameters include mineralization, mineral orientation, collagen cross-links, and microcracks. Because dentin shares most of these parameters with bone, evaluating the mechanical strength of dentin by such structural and quality parameters seemed reasonable. In the case of dentin, structural parameters could include the number of dentinal tubules and the degree of occluded tubule lumens; quality parameters would include mineral density, apatite orientation and AGE content.

The purpose of this study was to investigate the mechanical strength of human dentin in terms of structural and quality parameters, and to identify the factors most influential in regulating its mechanical strength.

## Results

### Flexural strength and toughness

**Figure 1:**
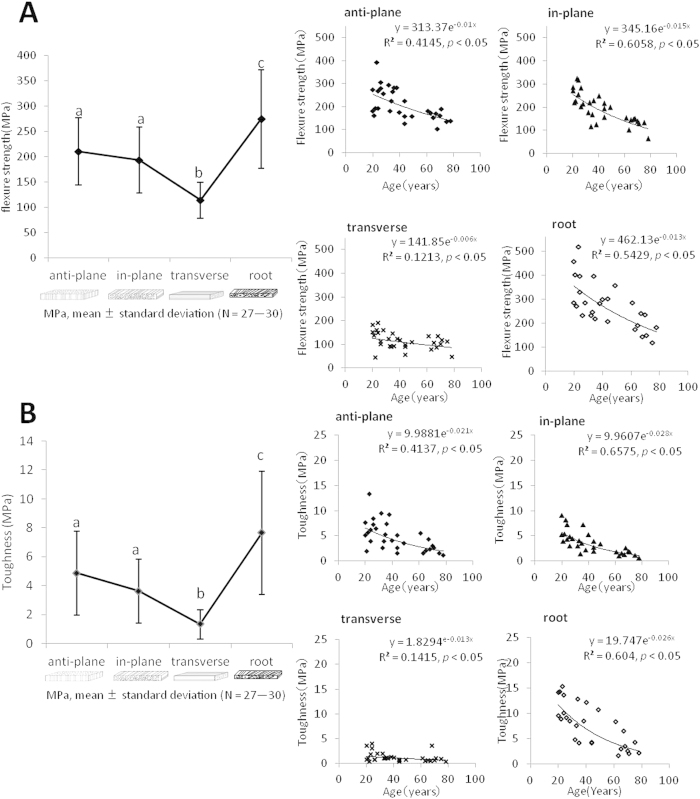
Flexural strength (A) and toughness (B) of human crown and root dentin with different tubule orientations and correlation with age. (**a**–**c**) There were no statistically significant differences between groups indicated by the same letters (two-factor ANOVA, Tukey’s HSD test, *p* < 0.01).

Flexural strengths of crown dentin in the anti-plane longitudinal, in-plane longitudinal, and transverse groups were 210 ± 67, 193 ± 65 and 114 ± 36 MPa, respectively, and that of root dentin was 274 ± 97 MPa. Transverse specimens showed significantly lower fracture strength compared with other groups; while root specimens showed higher fracture strength (*p* < 0.01, Fig. 1A left). In all the tested groups, the flexural strength decreased with aging (Fig. 1A right).

Toughnesses in the anti-plane longitudinal, in-plane longitudinal, transverse, and root groups were 4.9 ± 2.9, 3.6 ± 2.2, 1.3 ± 1.0, and 7.7 ± 4.3 MPa, respectively. Toughness showed a tendency similar to that of flexural strength. The transverse group showed significantly lower toughness compared with other groups (*p* < 0.01) (Fig. 1B left). Toughness in all groups with different tubule orientations decreased with aging (Fig. 1B right).

The over 40 age group exhibited significantly lower flexural strength and toughness than the younger group (*p* < 0.05) (Supplementary Information).

### Relationship between flexural strength and mineral density, dentinal tubule density, and degree of occluded tubule lumens

**Figure 2:**
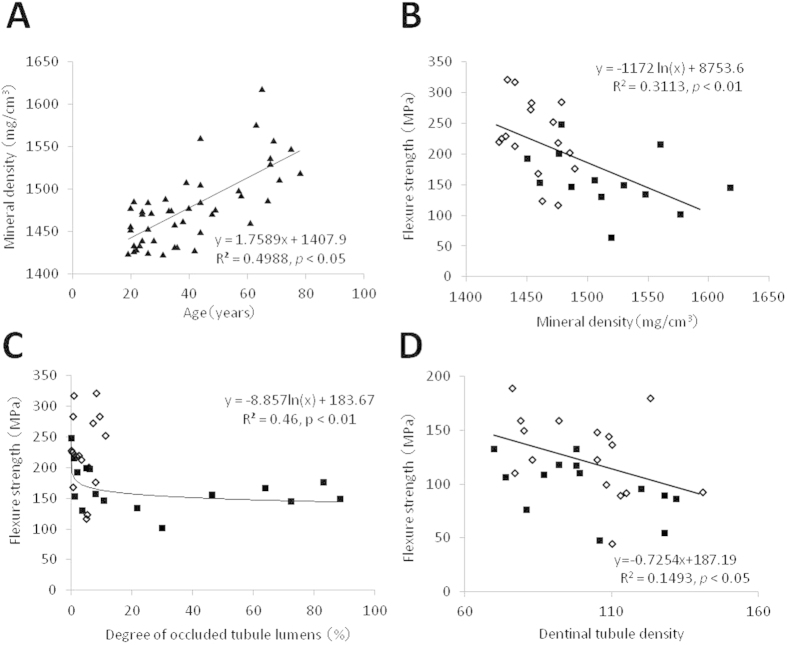
Mineral density of crown dentin in the in-plane longitudinal group taken from individuals aged 19–78 years (A). Effects of mineral density (**B**) and degree of occluded tubule lumens (**C**) on the flexural strength of crown dentin. Effect of dentinal tubule density (the number of dentinal tubules/48 × 64 μm^2^) on the flexural strength of human crown dentin in the transverse group (**D**). (open diamonds: age < 40 years, solid squares: age ≥ 40 years in (**B**–**D**)).

The mineral density of crown dentin was 1479 ± 45 mg/cm^3^ and increased with aging (*p* < 0.05) (Fig. 2A). Dentin with high mineral density exhibited low flexural strength, and this tendency was more marked in the group aged over 40 (Fig. 2B). The group aged under 40, which recorded low mineral density, demonstrated a low degree of occluded tubule lumens; while the aged group, which exhibited high mineral density and a high degree of occluded tubule lumens, demonstrated low flexural strength (Fig. 2C). Aged dentin with a high degree of occluded tubule lumens resulting in higher mineral density exhibited lower flexural strength than young dentin with a low degree of occluded tubule lumens. Dentin with a high density of dentinal tubules tended to have low flexural strength (Fig. 2D).

### Hardness and Young’s modulus

**Figure 3:**
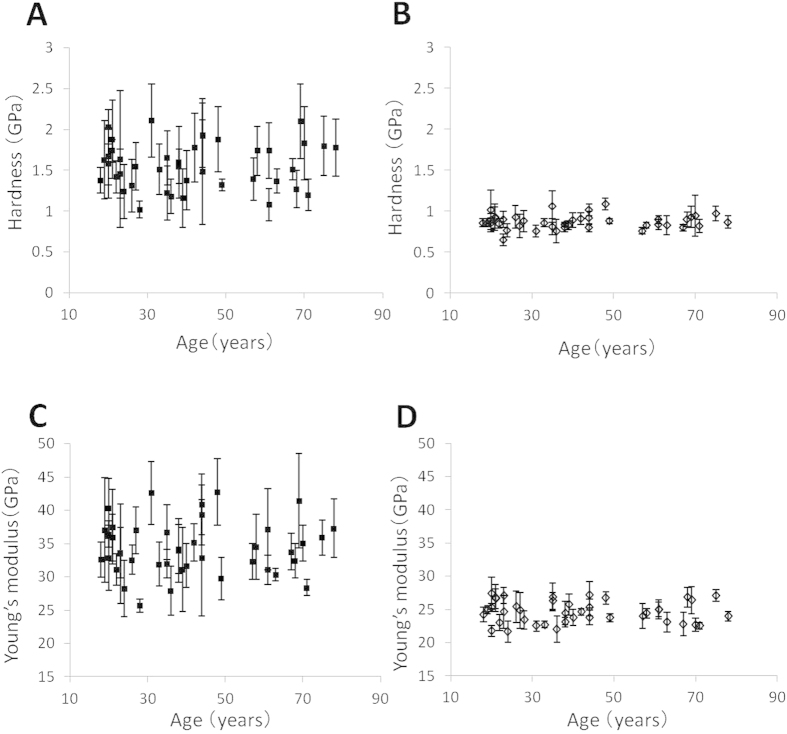
Hardness and Young’s modulus of human crown dentin taken from individuals aged 19–78 years. (**A**,**C**) peritubular dentin; (**B**,**D**) intertubular dentin.

The micro hardness of peritubular dentin was 1.6 ± 0.3 GPa and that of intertubular dentin was 0.9 ± 0.1 GPa (Fig. 3A,B). The Young’s moduli of peritubular dentin and intertubular dentin were 34.9 ± 3.9 and 24.8 ± 1.6 GPa, respectively (Fig. 3C,D). The hardness and the Young’s modulus of both peritubular and intertubular dentin did not change with aging (Fig. 3).

### Apatite orientation

**Figure 4:**
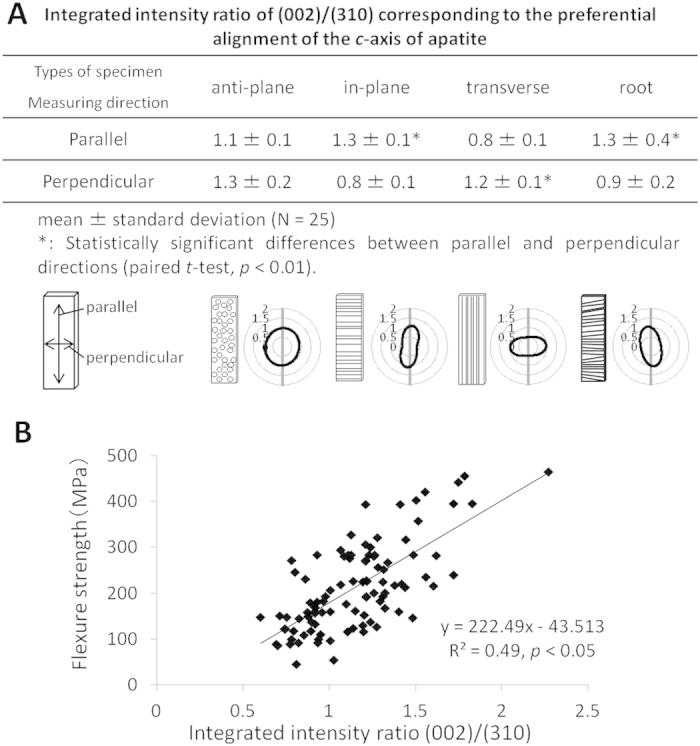
Distribution of the *c*-axis of apatite in human crown and root dentin with different tubule orientations (A). Effects of the apatite orientation on the flexural strength of human crown and root dentin (**B**).

Based on the X-ray diffraction data of the specimens parallel and perpendicular to the long axis, the integrated intensity ratios of the parallel direction in the in-plane longitudinal and root groups were significantly higher than those of the perpendicular direction. Perpendicular direction in the transverse group was significantly higher than the parallel direction (*p* < 0.01) (Fig. 4A). In the anti-plane longitudinal group, the integrated intensity ratio did not show a significant difference between the parallel and perpendicular directions. This revealed that the preferential alignment of the *c*-axis of apatite was perpendicular to the dentinal tubule orientation. The flexural strength significantly increased in specimens with strong alignment of their apatite *c*-axis (*p* < 0.05) (Fig. 4B).

### AGEs content

**Figure 5:**
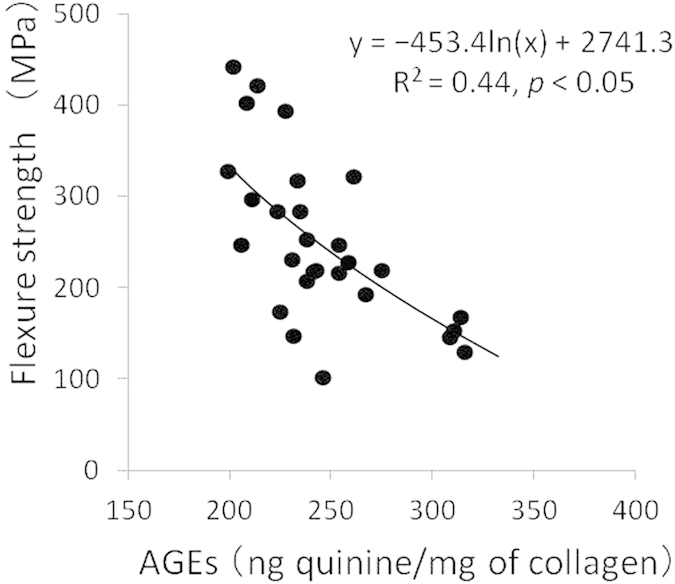
Quantity of advanced glycation end-products (i.e., pentosidine) in human dentin collagen in relation to flexural strength.

A significant negative correlation was found between flexural strength and the pentosidine content of both crown and root dentin (*p* < 0.05) (Fig. 5).

### Multiple linear regression analysis

Age was identified as the most influential factor affecting the flexural strength of dentin by the least corrected Akaike information criterion. Further analysis was performed to identify which were significant factors in the aging of dentin. In the least squares regression analysis, AGEs content and mineral density were influential as the quality parameters; while the degree of occluded tubule lumens was influential as the structural parameter (Table 1).

## Discussion

The variance inflation factor indices of both quality and structural parameters were below 1.5 (Table 1), indicating that the analysis was not affected by the multicollinearity. The least squares regression analysis revealed that both mineral density and AGEs content were important quality parameters accounting for the mechanical strength of dentin; these have been well acknowledged as influential factors for regulating bone strength^28^,^29^,^30^. The degree of occluded tubules was also revealed as a significant structural parameter for regulating dentin strength. This is reasonable because the occluded tubules can lead to rapid crack propagation^5^.

The results showed that the flexural strength of the transverse group was significantly lower than that of the others (Fig. 1A left). Arola *et al*.^18^ previously investigated the effects of tubule orientation on flexural strength, and reported that the flexural strength of the anti-plane longitudinal and transverse groups was 160 ± 22 and 109 ± 10 MPa. In the present study, the flexural strength of these groups was 210 ± 67 and 114 ± 36 MPa, respectively, showing a similar tendency of anisotoropy to that reported by Arola *et al*. The difference between the data in these two studies may be the result of the different geometries of the bending tests.

In the anti-plane longitudinal group, which showed high flexural strength, the collagen fibrils distributed in the planes perpendicular to the tubules might be influential in effectively resisting crack propagation leading to fracture.

The present study showed the low flexural strength of aged dentin in all groups with different tubule orientations (Fig. 1A right and Appendix). The post-yielding strain of all the specimens was markedly low in the stress-strain curve for flexural testing, and this explained the brittleness of the dentin specimens. Toughness thus showed a similar pattern to flexural strength (Fig. 1B). The flexural strength and toughness of root dentin showed large standard deviations, because regulation of the tubule orientation of root dentin was difficult for the curved, uneven configurations of its dentinal tubules. For future experiment, the dentin specimens should be selected by microscopic or CT observation to check the tubule orientations and micro flaws such as crack and caries.

The hardness and Young’s modulus of intertubular and peritubular dentin did not change with aging despite the high mineral density and high degree of occluded tubule lumens in the aged specimens (Fig. 3). This is because mineralization with aging is mostly because of the occlusion of dentinal tubule lumens with calcified materials rather than the mineralization of intertubular and peritubular dentin. The occlusion of dentinal tubule lumens did not affect the hardness and the Young’s modulus at the nano level, but might affect them at the micro level.

In a crack propagation test of human dentin^8^, performed *in situ* by a static three-point bending test under observation with an environmental scanning electron microscope, unfilled tubules tended to initiate microcracks prior to the main crack penetrating the tubule, resulting in crack deflection and branching. Unfilled tubules in young dentin had significantly more microcracked tubules in the crack wake compared with aged dentin, which can act as uncracked-ligament bridging. Conversely, occluded tubules in aged dentin showed less microcracking. At an early stage, a crack propagates around the peritubular cuff and does not penetrate the tubules. At a later stage, a crack propagates with less deflection and penetrates into occluded tubules. Less microcracking in aged dentin would lead to less ductility and reduced uncracked-ligament bridge formation^8^; therefore, aged dentin demonstrates low fracture resistance. In the present study, in which dentin with a high degree of occluded tubule lumens showed low flexural strength (Fig. 2C), the same mechanism of crack propagation may explain the lower fracture resistance of aged dentin. Other study of fatigue fracture testing also revealed a similar mechanism of microcracking and crack bridging as explanation of the low fracture resistance of aged dentin^6^.

In the present study, the apatite crystals of dentin were shown to align perpendicular to the dentinal tubules (Fig. 4). Because the apatite in the cortical bone aligns parallel to the orientation of collagen fibrils^31^,^32^,^33^, the apatite may align along the felt-like structure of collagen fibrils perpendicular to the tubule orientation. Strong alignment of dentin apatite may contribute to the anisotropy of the flexural strength of dentin according to the tubule orientation, since apatite aligned along the collagen fibrils can reinforce the intertubular dentin resulting in increased fracture resistance. The anisotropy of dentin and cortical bone in relation to their tubule structure was found to be different. Because the Haversian canals, which are microstructures in the cortical bone, align parallel to collagen fibrils in long bones, the Young’s modulus of the long bone was high in the direction parallel to the Haversian canals^34^,^35^,^36^,^37^.

Collagen cross-links can be divided into lysyl hydroxylase and lysyl oxidase-mediated enzymatic immature divalent cross-links, mature trivalent pyridinoline and pyrrole cross-links, and AGEs induced by non-enzymatic glycation or oxidation reaction, such as glucosepane and pentosidine^38^,^39^,^40^,^41^. Recently, collagen enzymatic and non-enzymatic cross-links in bone have been shown to affect not only the mineralization process but also microdamage. Saito *et al*.^42^,^43^ examined the BMD and the content of enzymatic and non-enzymatic cross-links of cortical bone in sound and osteoporosis patients with femoral neck fractures. The results suggested that the low levels of BMD and enzymatic collagen cross-links, as well as excessive formation of AGEs, could be the cause of low bone strength in osteoporosis patients^44^,^45^,^46^,^47^,^48^,^49^. Based on these findings, the quality and quantity of collagen cross-links have been widely accepted as significant factors regulating the mechanical properties of bone^38^,^50^,^51^. Furthermore, serum or urine pentosidine levels are now being used to estimate future fracture risk in osteoporosis and diabetes^52^,^53^,^54^,^55^. Miura *et al*.^27^ confirmed by immunohistochemical analysis that CML, which is an AGE, accumulated in the collagen fibrils around the dentinal tubules in aged dentin.

In the present study, aged dentin with a high level of pentosidine was shown to have low flexural strength (Fig. 5), because aged dentin collagen becomes more fragile on account of the accumulated AGEs. This can be considered as one of the reasons for the lower mechanical strength of aged dentin.

In conclusion, our study confirms that the flexural strength and toughness of human dentin decreases with aging. High mineral density due to occlusion of the dentinal tubules and accumulation of AGEs in dentin collagen were identified as the primary causes for the low mechanical strength of aged dentin.

The unchanged modulus with aging may be explained because calcification occurs within the tubule and not in the peritubular or intertubular dentin. Dentin with high mineral density due to occlusion of the dentinal tubules showed low flexural strength probably due to less resistance against crack propagation.

Aged dentin collagen becomes more fragile because of accumulated AGEs. This indicates, as proved in bone, that the organic components influence the strength of dentin.

## Methods

All experiments were carried out in accordance with protocols approved by the Ethics Committee of Osaka University (H21-E29).

### Preparation of dentin specimens

**Figure 6:**
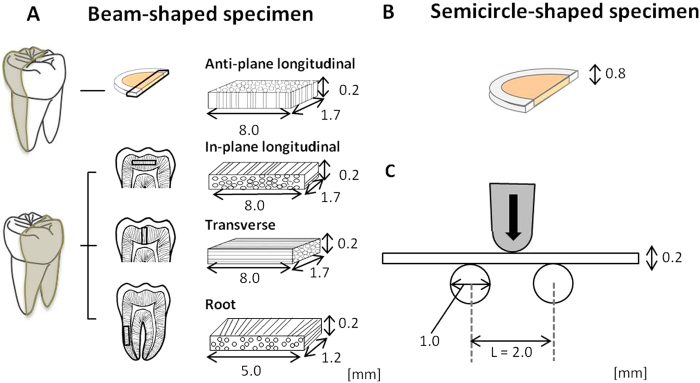
Configuration of human dentin specimens and geometry of flexural fracture testing. Beam-shaped specimens of crown dentin with three different dentinal tubule orientations and root dentin (**A**), a semicircular specimen (**B**). Beam-shaped specimens were subjected to flexural fracture testing using three-point bending geometry (**C**). Appendix: Flexural strength and toughness of human crown and root dentin with different tubule orientations in the aged group (age ≥ 40; black bars; N = 14 and the young group (age < 40; white bars; N = 16). *Statistically significant differences between the aged and young groups (*t*-test, *p* < 0.05).

Freshly extracted human molars (n = 46), whose origin and background including age, gender, type of tooth, and reasons for extraction could be identified, and which were free from fractures and caries, were selected. The teeth were stored in Hanks’ balanced salt solution (HBSS) at 4 °C and used within 3 months of extraction. The protocols used were approved by the Ethics Committee of Osaka University (H21-E29) and informed consent was obtained from all subjects. Beam-shaped specimens of crown dentin with three different dentinal tubule orientations, which measured approximately 1.7 × 0.2 × 8.0 mm, were sectioned with a low-speed diamond saw (IsoMet 2000, Buehler, IL, US) and adjusted with emery papers (#1500, 2000, Buehler) and a buffing cloth with a polisher (EcoMet, Buehler). Three tubule orientations were present (Fig. 6A). In transverse orientation specimens, the tubule orientation was parallel to the long axis. In specimens with in-plane longitudinal orientation, the tubule orientation was perpendicular to the long axis and parallel to the short axis; and in anti-plane longitudinal specimens, the tubule orientation was perpendicular to both the long axis and short axis. A root specimen measuring approximately 1.2 × 0.2 × 5.0 mm was also regulated by the long axis of the specimen parallel to the tooth axis (Fig. 6A). These crown and root specimens were obtained from one tooth. Semicircular specimens of crown dentin, sectioned parallel to the tooth axis with a thickness of approximately 0.8 mm, were also obtained from the same tooth (Fig. 6B).

### Measurement of mineral density

Beam-shaped specimens of crown dentin were scanned using peripheral quantitative computed tomography (CT; XCT Research SA, Stratec Medizintechnik, Germany). The specimens were kept fully hydrated in acrylic tubes during scanning. Each specimen was scanned longitudinally at three locations at intervals of 0.4 mm in crown dentin with a CT scanning speed of 10.0 mm/s and a voxel size of 0.08 mm. The average of the three scanned slices was taken as the mineral density of the specimen.

The mineral densities of the specimens were compared among different age groups by analysis of variance (ANOVA) and Sheffe’s *F*-test at a 95% level of confidence (StatView 5.0, Abacus Concepts, NJ, USA). The correlation between mineral density and age was analysed mathematically (Analysis ToolPak in Excel 2007, Microsoft, WA, USA).

### Measurement of flexural strength and toughness

Flexural fracture testing was conducted with beam-shaped specimens using three-point bending geometry (Fig. 6C) and using a universal testing machine (Autograph AG-IS, Shimadzu, Japan). Samples were loaded to failure under moist conditions with HBSS at ambient temperature with a crosshead speed of 0.1 mm/min. Flexural strength (σ, N/mm^2^) was calculated as: σ = 3*PL*/2*wt*^2^, where *P* (N) is the maximum load at fracture, *L* (mm) is the support span (*L* = 2 mm, constant), and *w* (mm) and *t* (mm) are the width and thickness of the specimen, respectively. Toughness (*u*, N/mm^2^) was also calculated from the stress–strain curve of the flexural testing: *u* = *U* (3/*L*) (*y*_max_^2^/*I*_*x*_), where *U* (N/mm) is the absorption energy to failure, and *y*_max_ (mm) is the distance of the surface from the neutral surface: y_max_ = *t*/2, and *I*_*x*_ (mm^4^) is the moment of inertia of the area: *I*_*x*_ = *wt*^3^/12.

The mechanical properties among individual specimens with different tubule orientations were compared by two-factor ANOVA followed by a Tukey’s honestly significant difference (HSD) test at a 95% level of confidence (PASW Statistics for Windows, Version 18.0, SPSS Inc Chicago, IL, USA). Specimens of different age groups were compared by one-factor ANOVA and Sheffé’s *F-*test at a 95% level of confidence with StatView 5.0. The correlation between mechanical properties and age was analysed mathematically with Analysis ToolPak.

### Calculation of dentinal tubule density and degree of occluded tubule lumens

Fractured surfaces of the transverse specimens after the flexural fracture testing were observed under a scanning electron microscope (SEM; JSM6390LV, JEOL, Japan) at a magnification of ×2000. Surfaces were sputter-coated with a gold–palladium alloy prior to observation. Dentinal tubule density was defined as the average number of dentinal tubules per unit area from five locations in the central opposite of the loading side in the SEM images. The degree (%) of occluded tubule lumens was calculated as the number of perfectly occluded tubule lumens divided by the total number of dentinal tubules.

The correlations between the flexural strength and dentinal tubule density and the degree of occluded tubule lumens were analysed mathematically with Analysis ToolPak.

### Measurement of hardness and Young’s modulus

Semicircular specimens were polished using emery waterproof abrasive papers (#1000, 1500, 2000, Sankyo-Rikagaku, Japan) and buffing cloths with alumina polishing suspension (1.0 CR, 0.3 CR, 0.05 CR, Baikalox CR, Baikowski, France). The specimens were ultrasonically cleaned in deionized water for 5 min to remove the debris on their surfaces. The dentin specimens were dried in air for 1 d at room temperature.

Nanoindentation was conducted by using a diamond Berkovich pyramidal indenter with a load-control-type nanoindentation system (ENT-1100a, Elionix, Japan). The maximum indentation load was 1 mN and the duration of the testing force was 180 s. The indentation was performed five times each for peritubular and intertubular dentin in a specimen, while observations were conducted using a charge-coupled device microscope attached to the nanoindentation system.

### Analysis of apatite orientation

After the fracture testing, beam-shaped specimens with all types of tubule orientations, stored in HBSS at 4 °C, were taken to analyse the distribution of the preferential alignment of the *c*-axis of biological apatite using a microbeam X-ray diffraction system equipped with a transmission optical system (R-AXIS BQ, Rigaku, Japan)^56^,^57^. Molybdenum-Kα radiation was generated at 50 kV and 90 mA (4.5 kW). The incident beam was collimated into a 300-μm circular spot by a double-pinhole metal collimator and radiated vertically onto the specimen. The X-ray diffraction data were recorded using an imaging plate (Fuji Film, Japan) for 300 s and the (002) and (310) reflections were identified in the X-ray profile. The diffraction data parallel and perpendicular to the long axis of the specimens were extracted (Fig. 4A) and the integrated intensity ratio of the (002) diffraction peak to the (310) diffraction peak was calculated. The intensity ratio corresponds to the degree of preferential alignment of the *c*-axis of the apatite crystallites^58^.

The integrated intensity ratios between the directions of the specimens parallel and perpendicular to the long axis were compared by paired *t*-test. The correlations between the flexural strength and integrated intensity ratios were analysed mathematically using Analysis ToolPak.

### Quantification of AGEs

Quantification of AGEs of dentin collagen was conducted as reported previously^59^. After the fracture testing, beam-shaped dentin specimens were pulverized in a liquid nitrogen-cooled freezer mill. The powder was demineralized with 0.5 M ethylenediaminetetraacetic acid (EDTA) in 50 mM Tris buffer (pH 7.4) for 96 h at 4 °C. The demineralized dentin residues were then sequentially suspended in potassium phosphate buffer (pH 7.6, ionic strength 0.15) and reduced at 37 °C with non-radioactive sodium borohydride (NaBH_4_). The reduced specimens were hydrolysed in 6N hydrochloric acid at 110 °C for 24 h. The resulting hydrolysates were then analysed for cross-links using high-performance liquid chromatography (HPLC) (LC9, Shimadzu) equipped with a cation exchange column (AA pack-Na, JASCO, Japan) linked to an online fluorescence flow monitor (RF10AXL, Shimadzu).

The amount of the AGE pentosidine, which is one of the cross-links produced by non-enzymatic glycation, was detected by natural fluorescence with excitation at 335 nm and emission at 385 nm. The quantity of the cross-links was expressed as ng quinine/mg of collagen.

The correlation between the number of cross-links and flexural strength was analysed mathematically with Analysis ToolPak.

### Multiple linear regression analysis

A multiple linear regression analysis was performed to identify the dominant factors that affect the mechanical strength of dentin using JMP-10 analytical software (SAS, NC, USA). In this study, stepwise regression and least squares regression were used. Flexural strength was identified as an objective variable, while explanatory variables were age, mineral density, number of dentinal tubules, degree of occluded tubule lumens, apatite orientation, and AGE content. All factors with *p*-values less than 0.05 were used to generate the stepwise linear regression model. The least corrected Akaike information criterion^60^ was used for presenting the statistically significant combination of parameters for predicting the objective variable. In addition, least squares regression was conducted to identify the influential explanatory variables to the objective variable in relation to quality and structural parameters.

## Additional Information

**How to cite this article**: Shinno, Y. *et al*. Comprehensive analyses of how tubule occlusion and advanced glycation end-products diminish strength of aged dentin. *Sci. Rep.* **6**, 19849; doi: 10.1038/srep19849 (2016).

## Supplementary Material

Supplementary Information

## Figures and Tables

**Table 1:** Least squares regression analysis on the quality and structural parameters influenced by aging.

	estimated coefficient	standard error	t-values	*p*-values	standard partial regression coefficient	variance inflation factor
(A) Quality parameters						
intercept	1839.20	340.75	5.40	0.0003	0	–
mineral density	−0.89	0.22	−3.99	0.0026	−0.63	1.02
apatite orientation	103.78	73.20	1.42	0.1867	0.26	1.44
AGEs content	−1.66	0.40	−4.10	0.0022	−0.76	1.42
(B) Structural parameters						
intercept	233.15	59.78	3.90	0.0005	0	–
degree of occluded tubule lumens	−0.98	0.42	−2.32	0.0272	−0.40	1.06
number of dentinal tubules	−0.24	0.57	−0.43	0.6709	−0.07	1.06
