# Supplementary material for: Comprehensive analyses of how tubule occlusion and advanced glycation end-products diminish strength of aged dentin
Source: Sci Rep. 2016 Jan 22;6:19849. doi: 10.1038/srep19849 (PMC4726429; doi:10.1038/srep19849)
Supplement: Supplementary Information [file srep19849-s1.pdf]

Comprehensive analyses of how tubule occlusion and AGEs diminish strength of aged dentin

Yuko Shinno, Takuya Ishimoto, Mitsuru Saito, Reo Uemura, Masumi Arino, Keishi Marumo, Takayoshi Nakano, Mikako Hayashi

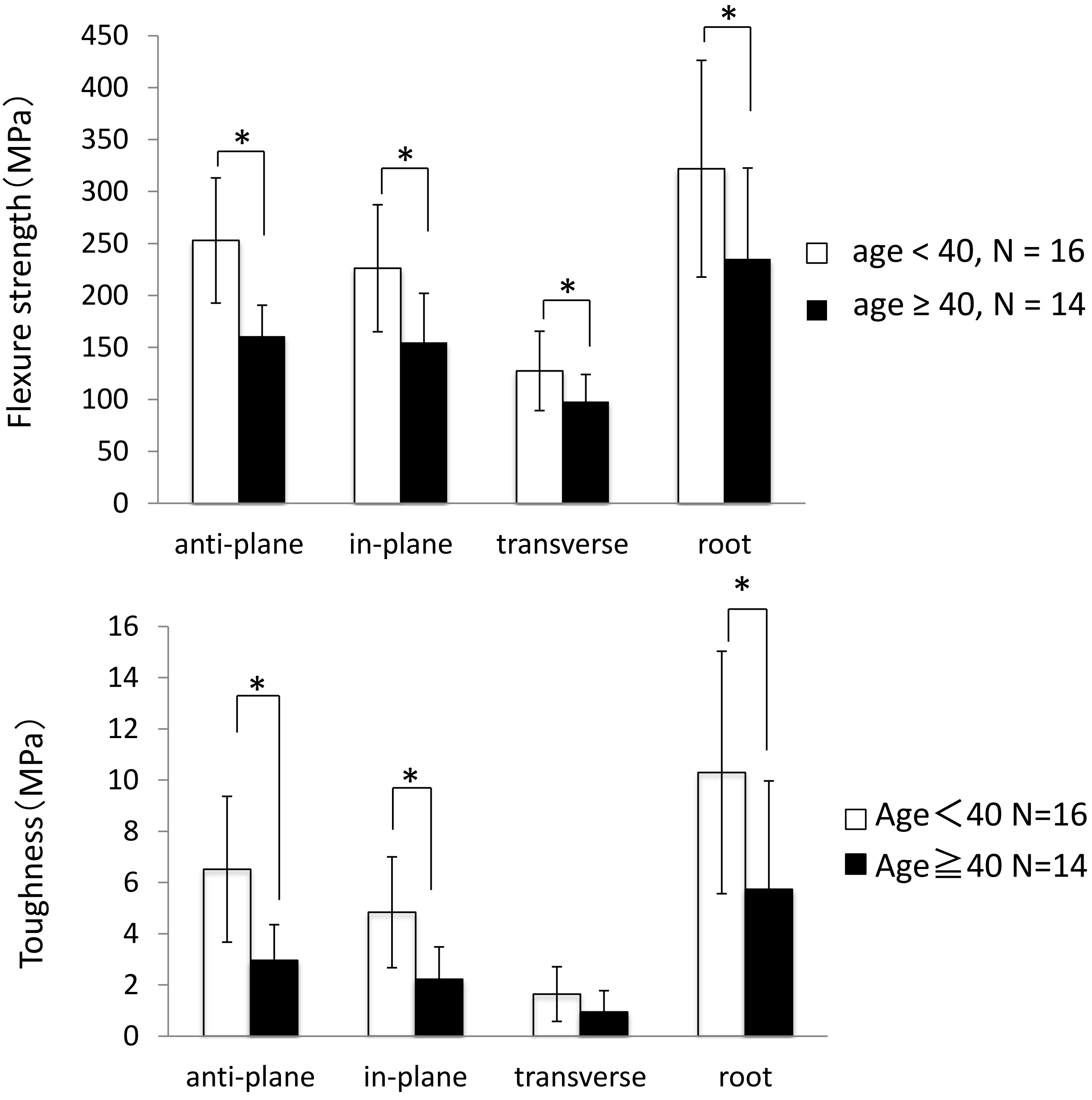

Appendix. Flexure strength and toughness of human crown and root dentin with different tubule orientations in the aged (age ≥ 40) and the young (age < 40) groups.

*: Statistically significant differences between the aged and young groups (*t*-test, $p < 0.05$).
